# Velocity loss is a flawed method for monitoring and prescribing resistance training volume with a free-weight back squat exercise

**DOI:** 10.1007/s00421-023-05155-x

**Published:** 2023-02-24

**Authors:** Ivan Jukic, Katarina Prnjak, Andrew King, Michael R. McGuigan, Eric R. Helms

**Affiliations:** 1 Sport Performance Research Institute New Zealand (SPRINZ), Auckland University of Technology, Auckland, New Zealand; 2 School of Engineering, Computer and Mathematical Sciences, Auckland University of Technology, Auckland, New Zealand; 3 School of Medicine, Western Sydney University, Sydney, Australia

**Keywords:** Velocity-based training, Resistance training, Fatigue, Exercise monitoring, Exercise prescription

## Abstract

**Purpose:**

The aim of this study was to examine the goodness of fit, prediction accuracy, and stability of general and individual relationships between velocity loss and the percentage of completed repetitions out of maximum possible (VL-%_repetitions_) in the free-weight back squat exercise. The effects of sex, training status and history, as well as personality traits, on the goodness of fit and the accuracy of these relationships were also investigated.

**Methods:**

Forty-six resistance-trained people (15 females and 31 males) performed a one-repetition maximum (1RM) test, and two repetitions to failure (RTF) tests, 72 h apart. RTF tests were performed with 70, 80, and 90% of 1RM with 10 min inter-set rest.

**Results:**

The findings question the utility of using general and individual VL-%_repetitions_ relationships to prescribe training volume with free-weight back squats as (1) the agreement in the %_repetitions_ completed until reaching a given velocity loss threshold across two consecutive testing sessions was unacceptable, regardless of the load used; and (2) the ability of general and individual VL-%_repetitions_ relationships to predict %_repetitions_ in a subsequent testing session were poor (absolute errors > 10%). Sex, training status and history, and personality traits did not affect the goodness of fit of general and individual VL-%_repetitions_ relationships or their prediction accuracy, suggesting potential generalisability of those findings among resistance-trained populations.

**Conclusions:**

VL-%_repetitions_ relationships do not seem to provide any additional benefits compared to costless, traditional methods and hence should not be used for monitoring and prescribing resistance training with a free-weight back squat exercise.

**Supplementary Information:**

The online version contains supplementary material available at 10.1007/s00421-023-05155-x.

## Introduction

Resistance training (RT) is recognised as an effective mode of training for inducing muscle strength, power, and hypertrophy adaptations. In addition, RT is often used for injury prevention and rehabilitation purposes and is also generally recommended due to its positive effects on general health and quality of life (Suchomel et al. 2016; Kraemer et al. 2002). There are numerous variables within a RT program (e.g., volume, frequency, intensity of load, exercise selection, movement velocity) that can be manipulated for the purpose of affecting acute responses, and subsequent adaptation, to RT. Of note, RT volume is considered a critical variable for driving neural and structural adaptations (Kraemer and Ratamess 2004; Schoenfeld et al. 2017). In traditional RT programs, set-volume is prescribed using a fixed, predetermined number of repetitions within the set based on the theoretical relationship between a maximum number of repetitions individuals can do with a given percentage of one-repetition maximum (%1RM). Indeed, this way of monitoring and prescribing RT set-volume is relatively simple and practical and can be managed with relative ease with large groups of people. However, large inter-individual variability exists for the maximal number of repetitions that can be completed to failure with different exercises and loads (Richens and Cleather 2014; Sánchez-Medina and González-Badillo 2011). This variability is problematic as it implies that prescribing the same relative load with a fixed number of repetitions will lead to heterogeneous training stimuli across individuals due to the potential differences in the level of effort experienced (i.e., number of repetitions left in reserve with respect to the maximum number of repetitions possible). Therefore, there is a need for alternative methods of monitoring and prescribing RT volume that take into account individuals’ performance capacities and provide a more homogeneous level of effort across individuals.

During an exercise set, as the number of repetitions performed with maximal voluntary effort increases, movement velocity progressively decreases due to fatigue accumulation. Indeed, findings of several studies indicate that monitoring velocity loss (VL) experienced during a set is an objective, practical and non-invasive indicator of the acute metabolic stress, hormonal response and mechanical fatigue induced by RT (González-Badillo et al. 2016; Pareja‐Blanco et al. 2017; Jukic et al. 2022). Moreover, a very close relationship between VL and the percentage of performed repetitions out of the maximum possible in a set (%_repetitions_) was observed for paused bench press and back squat exercises performed in a Smith machine with loads ranging from 50 to 85% of 1RM (González-Badillo et al. 2017; Rodríguez-Rosell et al. 2020). In addition, Gonzalez-Badillo et al. (2017) reported a low inter-individual variability in %_repetitions_ for a given magnitude of VL with 60% of 1RM. While these findings imply that this general VL-%_repetitions_ relationship can be used to monitor and prescribe RT set-volume, Sanchez-Moreno et al. (2021) recently reported higher goodness of fit for individual compared to general VL-%_repetitions_ relationship during the paused bench press exercise performed in a Smith machine and thus recommended the use of individual relationships which allow for a more homogeneous level of effort between individuals. Although these findings are useful, it is not clear whether the results are ecologically valid to free-weight exercises utilising the stretch–shortening cycle that involve vertical and horizontal barbell movements (Cotterman et al. 2005). Free-weight exercises utilising the stretch–shortening cycle are more popular among trainees, especially athletes, and have been shown to have a greater transfer of training effects to sports performance compared with concentric-only contractions, particularly in more complex multi-joint exercises (Bobbert et al. 1996; Stone et al. 2002). Therefore, it is necessary to examine the general and individual VL-%_repetitions_ relationships for free-weight exercises such as the barbell back squat and to determine the agreement between the %_repetitions_ on two consecutive testing sessions to ascertain the utility of such relationships for monitoring and prescribing RT using free weights.

When designing a RT program, sex, training status (e.g., strength levels), and history are typically considered due to their potential effects on adaptations to RT (Hunter 2014; Richens and Cleather 2014; James et al. 2018). However, the effects of these factors on the stability of the VL-%_repetitions_ relationship have not been comprehensively examined. In addition, since VL is used to monitor RT-induced fatigue and prescribe RT, personality traits such as emotional stability and conscientiousness could also affect the stability of VL-%_repetitions_ relationships due to their association with how individuals cope with fatigue (Calderwood and Ackerman 2011; De Vries and Van Heck 2002). Additionally, only second-order polynomial regression was previously used to model both general and individual VL-%_repetitions_ relationships. Thus, it is currently unknown whether simpler linear models could fit the data equally well and reduce the complexity of data analysis for practitioners seeking to establish these relationships. Finally, and most importantly, the predictive validity of both general and individual VL-%_repetitions_ relationships has not been examined to date. Evaluating those relationships on two different testing sessions and comparing goodness of fit is not enough when determining models’ utility in real-world settings. To consider a general or individual VL-%_repetitions_ relationship useful, they should demonstrate acceptable predictive validity first which could then be revaluated with individuals only sporadically, but not during every RT session.

Considering the above-mentioned scarcity in the literature, further examination of VL-%_repetitions_ relationships is clearly needed. Therefore, the purpose of this study was to (1) examine the goodness of fit of general and individual VL-%_repetitions_ relationships in a free-weight back squat exercise and determine the effects of sex, training status and history, as well as personality traits on the models’ fit; (2) quantify the agreement between the %_repetitions_ completed until reaching a given VL threshold on two testing sessions; and (3) determine the ability of general and individual VL-%_repetitions_ relationships to predict data in a subsequent testing session while also examining which factors affected the accuracy of the predictions. Such evidence is important to guide the VL approach to monitoring and prescribing RT with free-weight exercises.

## Materials and methods

### Design

This study employed a test–retest study design and is a part of a larger project investigating the validity of different velocity-based RT monitoring and prescription methods. Participants visited the laboratory on four separate occasions, separated by 48–72 h of rest between sessions. Participants first completed a familiarisation session that covered the free-weight back squat movement, the equipment and instruments used, an instruction to move the barbell up as fast as possible, and visual feedback on a screen indicating the velocity of the barbell. In the second session, participants completed an incremental loading test (i.e., 1RM) in the back squat exercise. In the last two sessions, participants completed a repetition to failure (RTF) test in the back squat, with 70, 80, and 90% of their pre-determined 1RM. All participants completed the experimental 1RM and RTF sessions at the same time of day (± 1 h) to avoid diurnal variation in exercise performance.

### Participants

Fifty-one strength-trained men and women (36 males and 15 females; 18 to 40 years of age) volunteered to participate in this study. However, five male participants withdrew from the study due to injury incurred at their work or recreational sporting activity not related to the study (*n* = 3) or for personal reasons (*n* = 2). Participants without a full data set were excluded from the analysis as the primary aim of the study was to determine the stability of the number of repetitions performed until a given VL threshold was met. Female and male participants recorded a 1RM relative to body mass in the free weight back squat of 1.25 ± 0.30 (range: 0.86–2.12) and 1.79 ± 0.35 (range: 1.18, 2.61), respectively. To be eligible for inclusion, participants confirmed that they (1) were willing to abstain from lower-body training during their participation in the study, (2) were not currently taking metabolic or cardiovascular function-altering medications, (3) were free of musculoskeletal injury, (4) were not actively taking anabolic steroids or had a history of anabolic steroid use, (5) had at least six months of RT experience training at least 2×/week, performing the back squat exercise at least 1x/week, with no more than two weeks in a row without RT during that period. Participants gave informed, written consent before commencing the study. The protocol of the current study was approved by the University Ethics Committee (approval number: 20/55).

### Familiarisation session

Participants arrived at the laboratory and completed a training history questionnaire regarding their habitual RT practices. Participants’ body mass and height were then recorded using an electronic column scale and wall-mounted stadiometer (Seca Ltd, Hamburg, Germany), respectively. Participants then completed a standardised warm-up, which consisted of cycling at 100 rpm for 5 min; dynamic stretching for 2 min; 10 bodyweight lunges and squats; and 10 squats with barbell only. Participants were familiarised with the instruction to lift the barbell up as fast as they can during the concentric muscle action and the provided visual feedback on a screen indicating the mean velocity of the barbell. Participants were also instructed to take at least a momentary pause between repetitions, but not to take more than 2 s between repetitions, with the feet remaining in contact with the floor for all repetitions (i.e., no jumping or lifting of the heels). Participants were asked to provide training logs for their most recent, heaviest back squat session and to conservatively estimate their 1RM. This information was used to inform the warm-up loads for the subsequent 1RM session. Participants then completed 3 repetitions at 20, 40, and 60% of their estimated 1RM, and then 10 repetitions at 60% of their estimated 1RM, to ensure familiarity with the instructions. Participants received visual feedback of barbell velocity during these practice repetitions and practised the instruction to move the barbell up as fast as possible and avoid pausing for more than two seconds between repetitions (i.e., standing phase of the squat). To facilitate a habitual squatting technique, participants were instructed to adopt and maintain a self-selected foot stance and eccentric tempo which were monitored across sessions. At the end of the session, participants confirmed they understood and felt comfortable with the study instructions and performed at least two sets with consistent repetition velocities (± 0.02 m/s).

### One repetition maximum session: Day 2

Participants arrived at the laboratory and completed the same standardised warm-up as in the familiarisation session. A 20-kg barbell (Rogue, Columbus, Ohio, USA) and calibrated weight plates (Eleiko, Halmstad, Sweden, EU) were used for the 1RM assessment. The protocol for the 1RM session consisted of 3 repetitions at 20, 40, and 60%; and 1 repetition at 80% and 90% of the estimated 1RM, followed by 1RM attempts (Jukic et al. 2020a, b). If a 1RM attempt was completed successfully, the load was increased 1 to 12.5 kg in consultation with the participant, until they could not complete an attempt or until the movement technique was compromised. A maximum of five 1RM attempts per participant were completed to establish the 1RM. Three- and four-minutes rest were provided for participants between submaximal sets and 1RM attempts, respectively (Jukic et al. 2020a, b). Participants adopted a self-selected foot stance and eccentric tempo as during the familiarisation session. Upon reaching the bottom of the squat, participants received instruction to lift the barbell up as fast as possible. Verbal encouragement was provided by researchers and visual feedback on barbell velocity was provided on a screen, for all trials. Participants were required to squat to a depth where the top of the thighs was at least parallel to the floor, as determined by the researchers, and a camera was positioned perpendicularly to the participant, for repetition to be considered successful.

### Repetitions to failure sessions: Days 3 and 4

The standardised warm-up that was completed in the familiarisation and 1RM sessions was completed by participants at the RTF sessions. Participants then completed 10, 5, 3, and 1 repetitions of the free-weight back squat exercise against 30, 50, 70, and 90% of the heaviest load that is going to be lifted that day (i.e., 90% of the established 1RM load), respectively. Participants were provided 3 min of rest between warm-up sets, and 4 min prior to the first set to failure. To minimise fatigue from the high-repetition 70% test from influencing the repetitions completed in the subsequent 80 and 90% tests, the RTF loads were not tested in a randomised order. Instead, participants performed RTF from highest to lowest load (i.e., 90% 1RM first, 80% 1RM second, and 70% 1RM third). The lifting instructions, verbal encouragement, and visual feedback standardisation used in the familiarisation and 1RM sessions were also used for all sets completed during the RTF sessions. RTF sessions were separated by at least 72 h.

### Data acquisition

The training history questionnaire is presented in Supplementary File I. Briefly, the questionnaire asked participants about (1) the number of repetitions they typically performed in training; (2) the loading intensity at which they usually train, (3) the number of repetitions left in reserve they usually have at the end of training sets, and (4) their RT experience in years. These questions were multi-choice; therefore, the responses were treated as categorical. The frequency of responses was inspected for each category (overall and within levels of outcome variables used in models) and these variables were recoded by merging several response options to avoid having less than 5 responses in more than 20% of cells (Field et al. 2012). Specifically, the number of repetitions performed containing three levels (1–5, 5–8, and > 8) and the intensity of load (< 70, 70–80, 80–90) were transformed into categorical variables containing three levels. The number of repetitions left in reserve (0–2 and 2–4) and RT experience (≤ 3 and > 3) were transformed into categorical variables containing two levels.

The mean velocity of all repetitions was recorded using a GymAware linear position transducer (LPT). This validated LPT (Banyard et al. 2017) was placed on both sides of the barbell and perpendicular to the position between the hands and the loaded barbell sleeves, per the manufacturer’s instructions. The end of the LPT cable was vertically attached to the barbell by a Velcro strap. This LPT measures the total displacement of its cable in response to changes in the barbell position. Changes in barbell position with respect to time is used to determine instantaneous velocity, which is also provided by the LPT’s software. The GymAware v2.8.0 app was used on a tablet (iPad, Apple Inc., California, USA) to transmit the data obtained from the LPT via Bluetooth. The LPT attached to the right side of the barbell was connected to a TV screen, which provided visual feedback indicating the mean velocity of the barbell after each completed repetition. The analysis in the current study was completed using data from this LPT. Additionally, the mean velocity of all completed repetitions was manually recorded and organised in a Microsoft Excel spreadsheet (Microsoft Corporation, Redmond, Washington, USA) during each session, to avoid issues with online cloud data storage or internet connection. The same two researchers completed this task throughout data collection to ensure consistency and accuracy. Using the mean velocity data, the magnitude of VL was calculated as a relative difference between the fastest and the last repetition performed in a set.

The 50-item International Personality Item Pool (IPIP) Big Five Personality Inventory was used as a validated assessment of the human personality (Goldberg 1992; Ehrhart et al. 2008). This inventory contains 50 questions, 10 of each assessing the Big Five personality dimensions (Agreeableness, Conscientiousness, Extraversion, Neuroticism, and Openness). A 5-point, Likert-type scale ranging from 1 (very inaccurate) to 5 (very accurate) was used to administer the IPIP items. Mean scores across personality dimensions were obtained by averaging the scores for each item. Only conscientiousness and emotional stability were retained for the analysis in the present study.

### Statistical analysis

Linear and second-order polynomial regression models were used to determine the individual and general VL-%_repetitions_ relationships. General relationships were determined separately for each load and testing session while pooling together the data from all participants, whereas individual relationships were obtained for each participant separately, on both testing sessions and with each load. The goodness of fit of general relationships was examined through the coefficient of determination (*R*^*2*^) and residual standard error (*RSE*) of the models, whereas medians and ranges of *R*^*2*^ and *RSE* were evaluated for individual VL-%_repetitions_ relationships.

Linear mixed-effects models with the Gaussian conditional distribution and identity link function were used to examine the influential factors on individual relationships’ *R*^*2*^ and *RSE* (i.e., model fits and model errors). For this purpose, the load (3 levels), training experience (2 levels) and practices (2 or 3 levels), relative strength, conscientiousness and emotional stability were all considered as fixed effects.

To assess the agreement across days between %_repetitions_ until reaching a given VL threshold (i.e., from 5 to 60% > in 5% increments), the true modified value varies method of the Bland–Altman analysis for multiple measures per participant (Bland and Altman 2007) was used for each load separately. An a priori equivalent margin of ± 10% of the repetitions completed until reaching a given VL threshold was used in the interpretation and evaluation of bias and the associated 95% limits of agreement (LoA). This criterion was chosen since more than a 10% difference would not be considered an improvement over traditional RT prescription methods such as stopping sets at a predetermined perceived number of repetitions in reserve. The method of variance estimates recovery (MOVER) was used to calculate the confidence intervals (CI) for LoA, as this considers the multiple, repeated measurements taken (Zou 2013). For LoA, two one-sided tests (TOST) were performed as the equivalence test, with an α-value of 0.1 and a 1─2α CI. The null hypothesis of TOST was that the two values were not equal. The null hypothesis was rejected where the 1–2α CI was contained in full of the ± equivalent margin, and in this instance, the two datasets (i.e., data from the two testing sessions) were considered equivalent (Schuirmann 1987). All assumptions of the Bland–Altman analysis were met.

The predictive validity of the general and individual VL-%_repetitions_ relationships was examined by using the models from the first testing session (i.e., general model with all participants’ data pooled and individual models of each participant) and fitting them to the data of the second testing session. Thereafter, general, and individual models’ errors were evaluated by calculating an absolute difference between the observed and predicted data in the second testing session. Models’ absolute error of less than 5% was deemed excellent, 5–10% acceptable, and more than 10% error not useful for monitoring and prescribing RT. To examine factors which influenced the absolute differences between observed and predicted data, linear mixed-effects models were used, as previously described. Finally, to confirm the robustness of these findings, generalised linear mixed-effects models, with a binomial conditional distribution and logit link function, were also used to examine factors affecting the probability of not exceeding an absolute prediction error of 10%.

For all mixed models, participants (*n* = 46) were treated as random effects to control for repeated measurements and the general variation between participants. Since both fixed and random effects were used, restricted maximum likelihood estimation was used for the evaluation of the linear mixed-effects models whereas maximum likelihood, with Laplace approximation, estimation was used for generalised linear mixed-effects models. The contribution of both fixed and random effects to the explanatory power of any of the explored models was examined using a likelihood ratio test, deviance statistic, and Akaike Information Criterion score, before selecting the final model to obtain the best fit while maintaining model parsimony. Importantly, the reduction of the model structure was always theoretically motivated and was done as a last resort. The statistical significance of fixed effects was examined by t-tests based on the Satterthwaite approximation or Wald *Z* tests for linear mixed-effects and generalised linear mixed-effects models, respectively. For linear mixed-effects models, predictors’ estimates and 95% CI were calculated and presented whereas for generalised mixed-effects models odds ratios with associated 95%CI were evaluated and presented to aid the interpretation of the findings. For categorical predictors with more than 2 levels, post-hoc tests were performed with Holm-Bonferroni correction. More details on models’ specifications and diagnostics can be found in the supplementary file II.

All statistical analyses were performed in R language and environment for statistical computing (version 4.2.0, The R foundation for Statistical Computing, Vienna, Austria) using the *SimplyAgree* (Caldwell 2022), *lme4* (Bates et al. 2015), and *emmeans* (Lenth et al. 2018) packages, models’ performance using the *performance* (Lüdecke et al. 2021) and *DHARMa* (Hartig 2020) packages, and preparation and visualisation of data using the *tidyverse* (Wickham et al. 2019) and *sjPlot* (Lüdecke 2018) packages. Custom-written R script and associated dataset are available at the Open Science Framework repository (URL: https://osf.io/3yuvr/).

## Results

**Fig. 1:**
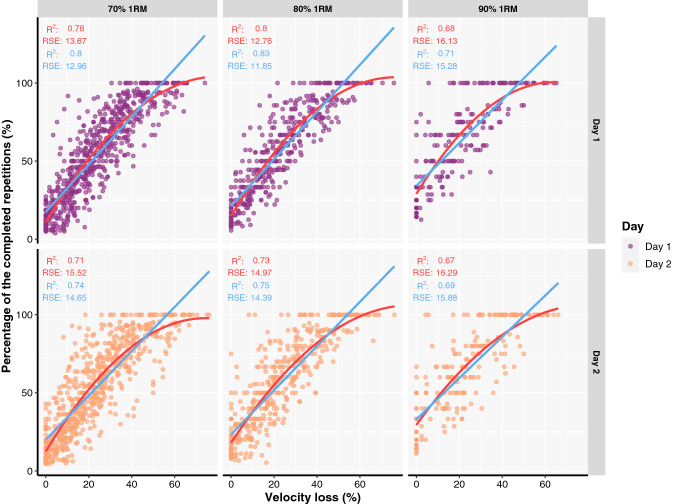
A general relationship between velocity loss and the percentage of the repetitions completed out of maximum possible fitted with linear (*red line*) and second-order polynomial regression (*light blue line*) models. Coefficient of determination (*R*^2^), as well as residual standard errors (RSE), are also presented for both linear (in *red*) and second-order polynomial regression (in *light blue*) models

**Fig. 2:**
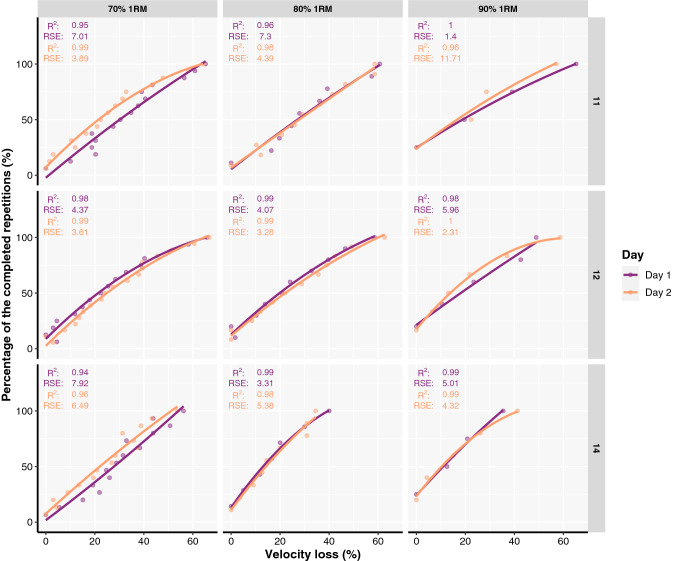
Individual relationships between velocity loss and the percentage of the repetitions completed out of maximum possible fitted with second-order polynomial regression models for representative participants. Coefficient of determination (*R*^2^), as well as residual standard errors (RSE), are also presented for both testing sessions. ID represents the participants’ identification number

The goodness of fit for the general VL-%_repetitions_ relationship across loads and testing sessions was generally strong and comparable for both linear and second-order polynomial regression models (Fig. 1; Table 1). However, the goodness of fit of the individual relationships was always stronger (Fig. 2; Table 2), regardless of the load, testing session and regression model.

**Table 1:** Percentages of the completed repetitions with respect to the maximum possible until reaching a given velocity loss threshold

VL	70% 1RM		80% 1RM		90% 1RM	
	Day 1	Day 2	Day 1	Day 2	Day 1	Day 2
VL5	19.05 ± 9.16	21.19 ± 11.73	27.72 ± 11.39	29.3 ± 18.25	49.05 ± 19.91	60.14 ± 22.51
VL10	30.47 ± 14.06	31.04 ± 13.59	41.83 ± 13.97	39.04 ± 17.29	45.5 ± 10.89	46.66 ± 14.1
VL15	40.96 ± 13.78	43.94 ± 14.39	45.75 ± 13.3	47.07 ± 17.56	62.41 ± 18.68	56.74 ± 17.53
VL20	51 ± 14.71	48.28 ± 15.5	56.61 ± 16.77	55.19 ± 17.6	74.83 ± 18.17	58.69 ± 17.98
VL25	58.28 ± 15.71	57.54 ± 14.19	66.27 ± 14.98	65.35 ± 14.77	71.26 ± 18.15	73.47 ± 15.87
VL30	69.83 ± 15.09	69.12 ± 17.3	73.85 ± 12.19	70.93 ± 14.06	77.86 ± 18.12	83.61 ± 16.66
VL35	77.96 ± 13.43	75.3 ± 14.61	82.71 ± 10.6	76.06 ± 15.92	88.89 ± 10.88	82.61 ± 18.41
VL40	82.77 ± 12.24	82.55 ± 14.22	85.04 ± 12.1	82.98 ± 7.87	95.41 ± 8.54	87.95 ± 14.91
VL45	90.12 ± 9.56	89.08 ± 7.08	89.5 ± 9.08	90.03 ± 9.15	98.21 ± 5.06	93.85 ± 11.39
VL50	90.34 ± 11.26	86.47 ± 15.52	95.74 ± 7.71	96.12 ± 5.88	97.96 ± 5.4	93.75 ± 12.5
VL55	91.87 ± 6.78	94.14 ± 8.95	96.63 ± 5.25	98.16 ± 4.11	100 ± 0	100 ± 0
VL60 ≥	96.63 ± 5.73	96.38 ± 4.92	100 ± 0	97.91 ± 5.9	100 ± 0	100 ± 0

*VL* velocity loss, *1RM* one-repetition maximum

**Table 2:** Goodness of fit for individual VL-%repetitions relationships established using linear and polynomial regression models on two testing sessions with different loads

Load	Model type	Day 1						Day 2					
		Median *R*^2^	Min *R*^2^	Max *R*^2^	Median RSE	Min RSE	Max RSE	Median *R*^2^	Min *R*^2^	Max *R*^2^	Median RSE	Min RSE	Max RSE
70% 1RM	Linear	0.93	0.35	0.98	8.32	4.79	25.33	0.90	0.52	0.99	9.73	2.89	21.46
70% 1RM	Polynomial	0.94	0.42	0.99	8.06	3.93	25.20	0.93	0.53	0.99	9.03	2.99	22.36
80% 1RM	Linear	0.93	0.60	0.99	8.41	2.37	21.48	0.92	0.43	0.99	9.35	3.23	26.24
80% 1RM	Polynomial	0.95	0.73	1.00	7.48	1.06	17.75	0.96	0.47	0.99	7.37	3.26	29.43
90% 1RM	Linear	0.96	0.24	1.00	7.58	0.12	41.10	0.92	0.49	1.00	10.81	1.85	33.71
90% 1RM	Polynomial	0.97	0.57	1.00	8.09	0.41	35.36	0.94	0.57	1.00	10.92	2.31	29.34

*1RM* one-repetition maximum, *R*^*2*^ coefficient of determination, *RSE* residual standard error

The goodness of fit of individual VL-%_repetitions_ relationships was affected by testing session, model type, and load (Table 3). *R*^2^ was higher in the second testing session (*p* < 0.001), when the second-order polynomial regression model was used (*p* < 0.001), and with 90% (*p* = 0.030) and 80% (*p* = 0.030) compared to 70% of 1RM load (Supplementary file III). Therefore, second-order polynomial regression models were used for later evaluation of the predictive validity of the VL-%_repetitions_ relationships.

**Table 3:** Factors affecting (1) the goodness of fit of individual VL-%_repetitions_ relationships; (2) absolute differences between predicted and observed %_repetitions_ in a subsequent testing session based on individual VL-%_repetitions_ relationships; and (3) the probability of individual VL-%_repetitions_ relationships exceeding a 10% prediction error

Predictors	Factors affecting R^2^			Factors affecting absolute differences			Factors affecting probability of exceeding 10% error		
	Estimates	CI	*p*	Estimates	CI	*p*	OR	CI	*p*
(Intercept)	0.97	0.77–1.16	< 0.001	10.95	−2.18–24.08	0.102	0.30	0.05–1.95	0.208
Day [Day 2]	−0.02	−0.04 to −0.01	0.006	–	–	–	–	–	**–**
Model [polynomial]	0.03	0.02–0.05	< 0.001	–	–	–	–	–	**–**
Sex [male]	0.04	− 0.02–0.10	0.235	−0.24	−4.24–3.75	0.905	0.98	0.76–1.27	0.885
Load [80% 1RM]	0.02	0.00–0.04	0.017	0.72	−0.49–1.93	0.245	1.90	1.36–2.64	< 0.001
Load [90% 1RM]	0.03	0.01–0.04	0.009	6.11	4.54–7.68	< 0.001	1.00	0.97–1.03	0.945
Emotional Stability	-0.00	− 0.00–0.00	0.433	−0.05	−0.27–0.17	0.643	1.02	0.98–1.05	0.370
Conscientiousness	− 0.00	− 0.01–0.00	0.316	0.11	−0.13–0.34	0.365	0.98	0.76–1.27	0.885
Training experience [> 3 years]	0.04	− 0.00–0.09	0.070	−0.09	−3.22–3.04	0.955	0.79	0.51–1.23	0.296
Loads practices [70–80% 1RM]	0.02	− 0.04–0.07	0.616	−3.09	−7.10–0.91	0.130	0.72	0.44–1.20	0.209
Loads practices [> 80% 1RM]	0.04	− 0.03–0.11	0.235	−5.48	−10.03–− 0.93	0.018	0.89	0.51–1.54	0.667
Repetitions practices [8–12 repetitions]	0.02	− 0.03–0.08	0.404	−1.10	−4.64–2.44	0.542	0.80	0.46–1.42	0.452
Repetitions practices [> 12 repetitions]	−0.00	− 0.06–0.06	0.984	−0.12	−4.02–3.78	0.953	0.51	0.27–0.98	0.043
Repetitions in reserve practices [> 2 RIR]	0.02	− 0.03–0.06	0.490	−1.82	−4.96–1.32	0.256	0.85	0.54–1.33	0.477
Relative strength (1RM/BM)	−0.04	− 0.10–0.03	0.255	1.17	−3.27–5.62	0.605	1.87	1.00–3.51	0.051
Random effects									
σ^2^	0.01			97.68			3.29		
τ_00_ _ID_	0.00			15.53			0.22		
ICC	0.30			0.14			0.06		
N _ID_	46			46			46		
Observations	552			1295			1295		
Marginal *R*^2^/conditional *R*^2^	0.139/0.393			0.072/0.200			0.049/0.106		

Note. Reference groups were the following: Load [70% 1RM], Sex [female], Training experience [< 3 years], Loads practices [< 70% 1RM], Repetitions practices [< 8 repetitions], Repetitions in reserve practices [< 2 RIR]. 1RM, one repetition maximum; BM, body mass; RIR, repetitions in reserve; OR, odds ratio; ICC, interclass-correlation coefficient; R^2^, coefficient of determination; CI, 95% confidence intervals; p, p value; VL-%_repetitions_, relationships between velocity loss and the percentage of the completed repetitions with respect to the maximum possible

**Fig. 3:**
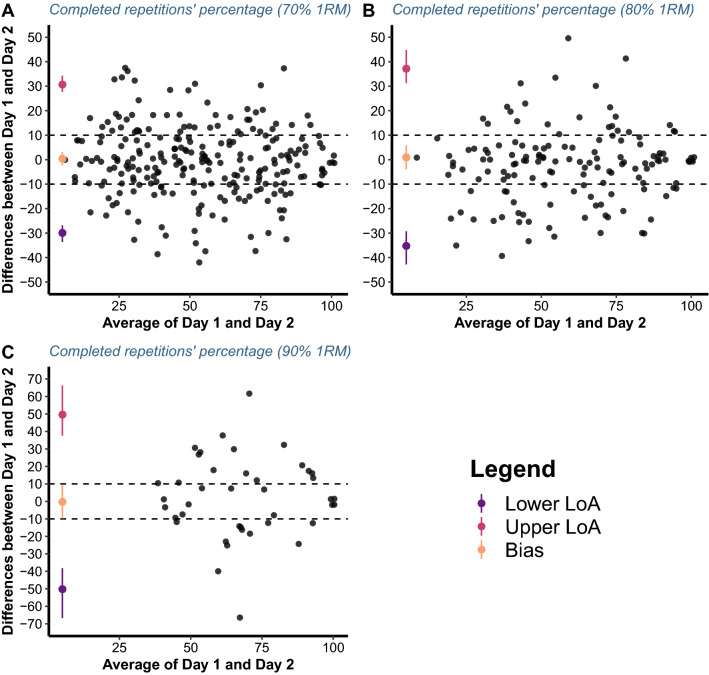
Bland–Altman plots illustrating the agreement between the percentages of the repetitions completed—out of maximum possible—until reaching a given velocity loss threshold on two testing sessions for 70 (**A**) 80 (**B**) and 90% of 1RM load (**C**). Dashed lines represent an equivalent margin of ± 10% whereas LoA represents limits of agreement

The null hypothesis for the equivalence of the %_repetitions_ until reaching a given VL threshold on day 1 and day 2 was not rejected for any of the examined loads since the 1–2α CI of LoA were always completely outside the ± equivalent margin of 10% (Fig. 3).

The predictive validity of the VL-%_repetitions_ relationship was not acceptable, regardless of whether general or individual relationships were fitted to the data since the absolute error between observed and predicted data on the second testing session—while using the data of the first testing session to make predictions—was always higher than 10%, regardless of the load used (Supplementary file IV). The linear mixed-effects model investigating factors affecting absolute differences between observed and predicted data on the second testing session revealed only load and training load practices were influential factors (Table 3), with absolute errors being lower with 90% compared to 80% and 70% of 1RM (*p* < 0.001). After a multiple comparison correction was applied, there were no differences between participants who often use very high (i.e., 90%) compared to those who use lower loads (i.e., 80 or 70% of 1RM) during their own training (*p* ≥ 0.071). Similarly, the generalised linear mixed-effects model examining factors affecting the probability of not exceeding an absolute prediction error of 10% also revealed only load and training load practices were influential factors (Table 3), with the probability of exceeding the 10% prediction error increasing with 90% compared to 70% (*p* < 0.001) and 80% of 1RM (*p* < 0.001) loads. There were no differences between participants who have different training load practices when multiple comparison correction was applied (*p* ≥ 0.130).

## Discussion

To our knowledge, this is the first study that examined the goodness of fit, prediction accuracy, and stability of general and individual VL-%_repetitions_ relationships in a free-weight back squat exercise while also exploring the effects of sex, training status and history, as well as personality traits on the goodness of fit and accuracy of those relationships. The main findings of this study were (1) individual rather than general VL-%_repetitions_ relationships yielded a higher goodness of fit for all loads and on both testing sessions; (2) goodness of fit for both individual and general relationships was higher on the second testing session compared to the first and with higher (i.e., 90 and 80% 1RM) compared to the lower loads (i.e., 70% 1RM), but was not affected by sex, training status and history nor personality traits; (3) for individual VL-%_repetitions_ relationships, second order polynomial regression models yielded a better goodness of fit compared to linear models whereas both regression models fit the data equally well for the general VL-%_repetitions_ relationship; (4) individual, but not general, VL-%_repetitions_ relationships displayed acceptable *RSE* apart with 90% 1RM in the second testing session; (5) the agreement between the %_repetitions_ performed across VL thresholds on two testing sessions was not acceptable, regardless of the load used; and (6) both general and individual VL-%_repetitions_ relationships established in the first testing session did not provide acceptable predictions of %_repetitions_ (due to high absolute errors) in the second testing session, and this prediction accuracy was not affected by sex, training status, history, or personality traits. Therefore, the findings of the present study question the utility of the VL-%_repetitions_ relationship to monitor and prescribe RT volume with a free-weight back squat exercise.

Researchers have previously reported high goodness of fit for the general VL-%_repetitions_ relationships with a wide range of exercises such as bench press (*R*^2^ ≥ 0.8), back squat (*R*^2^ ≥ 0.93), and shoulder press (*R*^2^ ≥ 0.99) (Hernández-Belmonte et al. 2021; Rodríguez-Rosell et al. 2020). In the present study, much lower values of *R*^*2*^ were observed (*R*^2^ = 0.67–0.80). This discrepancy between the findings could partially be explained by the mode of the exercise used. Previously reported data on the goodness of fit of general VL-%_repetitions_ relationships came from exercises performed in a Smith machine whereas participants in the present study performed a free-weight back squat exercise. Therefore, it seems that there is a higher variability between the individuals in %_repetitions_ completed until reaching a given VL threshold when free-weights are used compared to Smith machines. More importantly, individual VL-%_repetitions_ relationships always provided better goodness of fit compared to general VL-%_repetitions_ relationships. This finding agrees with the study by Sanchez-Moreno et al. (2021) who also recently reported higher goodness of fit for individual (*R*^2^ = 0.97–0.99) compared to general VL-%_repetitions_ relationships (*R*^2^ = 0.80–0.94) for the Smith machine bench exercise. Furthermore, second-order polynomial regression models always yielded a better fit to the VL-%_repetitions_ data compared to linear models, suggesting they should be used in practice. While these results are useful, it should also be noted that the RSE for both general (*RSE* ≥ 12.76) and individual (median *RSE* ≥ 7.37) VL-%_repetitions_ relationships always exceeded the 5% mark. This is important to consider as such an error—although being in an acceptable range for individual VL-%_repetitions_ relationships—may indicate that this prescription method does not provide any additional benefits for practitioners compared to more traditional RT prescription methods. These findings may also imply the instability of VL-%_repetitions_ relationships in providing accurate estimations of %_repetitions_ in practice. Finally, sex, training status and history as well as personality traits did not affect the goodness of fit of general and individual VL-%_repetitions_ relationships, indicating the potential generalisability of these findings.

Researchers previously recommended that each training set should be terminated as soon as a given VL is reached instead of prescribing a given, fixed, number of repetitions per set to be performed for each participant (Sánchez-Medina and González-Badillo 2011; González-Badillo et al. 2017). The rationale behind this recommendation was the strong VL-%_repetitions_ relationship for back squat and bench press exercises performed in a Smith machine. Indeed, descriptive data reported by Gonzalez-Badillo et al. (2017) indicate low inter-individual variability for the %_repetitions_ until reaching a given VL. However, Rodriguez Rosell et al. (2020) recently suggested that the prescription of RT volume by using VL should be specific for each exercise and load. Careful inspection of the %_repetitions_ until reaching a given VL data in the present study suggests that the variability between individuals may indeed be very high (i.e., high standard deviations), despite similar mean %_repetitions_ values for a given VL threshold between the two testing sessions. Furthermore, this variability remained when the descriptive data of the present study were broken down by sex, training experience, and relative strength (Supplementary file V). In fact, some differences could be observed, depending on the load and VL threshold, between sexes, participants with different levels of training experience, and relative strength. Nevertheless, it is important to highlight that the agreement between the %_repetitions_ until reaching a given VL on two testing sessions has not been examined to date. This is crucial as it directly assesses the practical utility of the VL-%_repetitions_ relationship for monitoring and prescribing RT set-volume. In this regard, the findings of the present study indicate substantial disagreement between %_repetitions_ performed until reaching a given VL threshold on two consecutive testing sessions (72 h apart), regardless of the load used. Collectively, due to considerable variability in the %_repetitions_ performed until reaching a given VL threshold across participants and the observed disagreement between %_repetitions_ for a given VL threshold on two consecutive testing sessions, VL-%_repetitions_ relationship does not appear to be stable enough for monitoring and prescribing RT volume with a free-weight back squat exercise.

Although many studies have been recently published on the VL-%_repetitions_ relationship, the predictive validity of this relationship has not yet been examined. Researchers have previously examined the goodness of fit of VL-%_repetitions_ relationships on two separate days (González-Badillo et al. 2017; Sánchez-Moreno et al. 2021). Since this goodness of fit was generally very high for bench press and back squat exercises performed in a Smith machine, the conclusion would be that VL-%_repetitions_ relationship can safely be used to reliably, and with high precision, prescribe and monitor RT set-volume. Unfortunately, these findings are of limited ecological validity for free-weight exercises and provide limited empirical support for the use of VL-%_repetitions_ relationships to monitor and prescribe RT set-volume. This is because practitioners cannot establish a VL-%_repetitions_ relationship in every single training session with their athletes, patients, or clients. Therefore, to confirm the usefulness of the VL-%_repetitions_ relationship, one would ideally want to know whether the established VL-%_repetitions_ relationship for a given individual during an initial testing session predicts future data, in this case, the %_repetitions_ completed in subsequent training sessions reasonably well. The findings of the present study indicate that the predictive validity of both general and individual VL-%_repetitions_ relationships was unacceptable since the mean absolute differences between the predicted and observed %_repetitions_ in the subsequent testing session always exceeded the 10% mark, regardless of the load used (Supplementary file V).

**Fig. 4:**
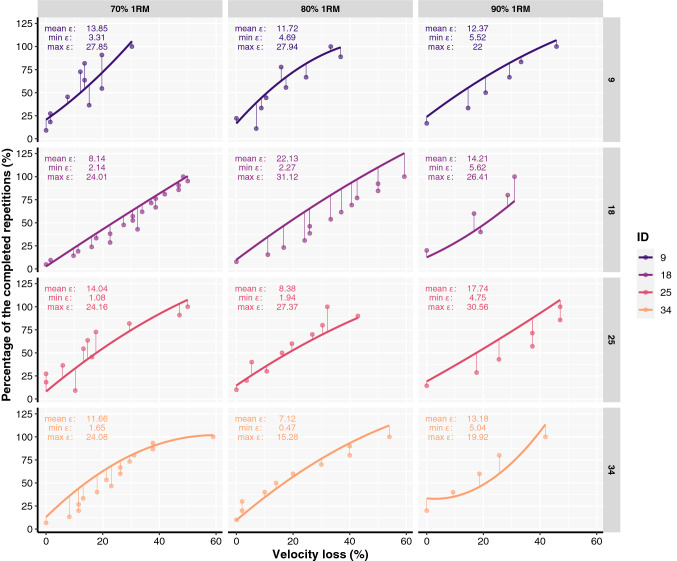
Individual relationships between velocity loss and the percentage of the repetitions completed out of maximum possible established with the second-order polynomial regression in the first testing session (line) and fitted to the data of the second testing session (dots). Thin vertical lines connecting thick lines and dots across participants represent distances (or residual errors) between predicted and observed data. Mean (mean *ε*), minimal (min *ε*), and maximal (max *ε*) errors are also presented for each participant and load. This data is from representative participants where models’ prediction errors always exceeded a 5% error mark and frequently exceeded errors of 10% or more

**Fig. 5:**
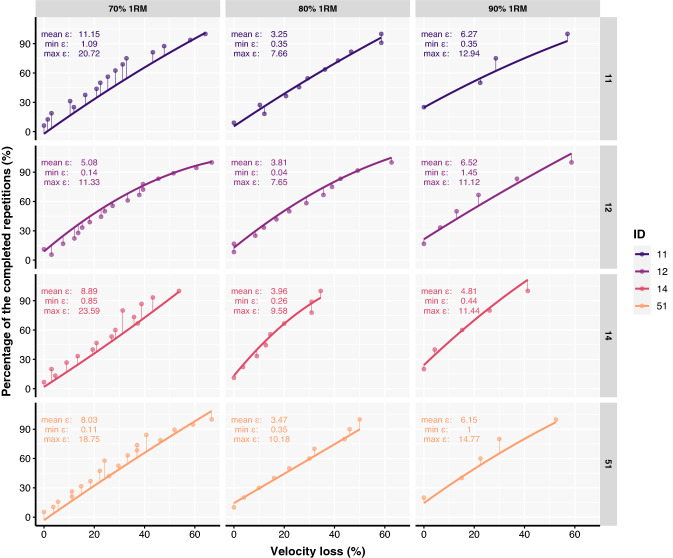
Individual relationships between velocity loss and the percentage of the repetitions completed out of maximum possible established with the second-order polynomial regression in the first testing session (line) and fitted to the data of the second testing session (dots). Thin vertical lines connecting thick lines and dots across participants represent distances (or residual errors) between predicted and observed data. Mean (mean *ε*), minimal (min *ε*), and maximal (max *ε*) errors are also presented for each participant and load. This data is from representative participants where models’ prediction errors did not always exceed the 5% error mark but still exceeded that or even the 10% threshold for at least one of the loads for every participant

On an individual basis, there may be some circumstances where VL could provide utility for volume prescription, but the model prediction error is often very high for some individuals, at least for some loads. For instance, Fig. 4 visualises representative participants’ data where models’ prediction errors always exceeded a 5% mark and frequently exceeded errors of 10% or more, as it was very often the case among the participants in the present study. In contrast, Fig. 5 demonstrates representative participants’ data where models’ prediction errors were often lower than 5% but still exceeded that or even the 10% threshold for at least one of the loads for every participant. A more nuanced look at these figures allows for an appreciation of the maximal prediction error for individual %_repetitions_ with a given VL threshold and load. This is important as only focusing on mean error across the %_repetitions_ can hide the inability of the model to accurately predict the entire VL-%_repetitions_ spectrum. This indicates that problems can arise when prescribing a specific VL with a specific load for an individual whose model’s prediction error was very high for that particular VL and its associated %_repetitions_.

The present study also aimed to investigate what factors are associated with the observed prediction errors and the probability of exceeding a 10% prediction error. Based on the results of both models, only the choice of load (i.e., 90% of 1RM) inflated the prediction error compared to lower loads. In addition, the loads participants used during their own training appeared to affect prediction errors, with those usually training with higher loads (i.e., > 80% of 1RM, on average) having lower prediction errors and thus lower probability of exceeding a 5% prediction error. However, training with higher loads, on average, could still imply somewhat higher confidence and motivation to increase muscle strength in general, which may or may not be related to the training experience. Finally, other training history factors, relative strength, sex, and personality traits did not affect prediction errors of VL-%_repetitions_ relationships. Thus, these findings could probably be generalised within the resistance-trained population with a free-weight back squat exercise.

The present study comprehensively examined the predictive validity of the VL-%_repetitions_ relationship for monitoring and prescribing RT while also exploring the effects of a range of factors related to RT. However, there are several limitations that should be considered when interpreting the results. Firstly, it is unknown whether the findings of the present study transfer to other free-weight exercises. However, it seems reasonable to posit that the effects of training history, and status, sex, and personality traits are likely applicable to a variety of exercises. Secondly, the participants in the present study had at least 6 months of RT experience; thus, it is unclear whether the current findings generalise to those without RT experience (e.g., sedentary populations, trainees completing other exercise modalities). This may partially explain why in the present study, training experience did not influence the outcomes of interest. Thirdly, since the loads were not performed in a randomised order, residual fatigue incurred from the heavier loads (i.e., 90 and 80% of 1RM) could have influenced performance in subsequent sets (i.e., 80 and 70% of 1RM) and the between-day agreement for the %_repetitions_ completed across VL thresholds. However, this potential influence was mitigated by at least 72 h rest between trials, long inter-set rest periods (i.e., 10 min), and the descending order of the load (i.e., higher to lower) in which participants completed the RTF sets. Fourthly, while efforts were made to recruit an equal sample of male and female participants, the number of females included in the analysis was substantially lower than males. However, it should be noted that the females in the present study had a wide range of strength levels, training experience, and different training practices, improving the sample’s generalisability, and perhaps explaining the lack of sex differences (Nimphius 2019) in this study. Finally, it should be acknowledged that the choice of the threshold for acceptable agreement and predictive validity could influence findings and interpretation thereof. However, the LoA for %_repetitions_ across testing sessions were much higher than 10% (even higher than 30%) and prediction errors rarely approached excellent or even acceptable levels. To conclude, a RT monitoring and prescription method with a higher than 10% error may not be justified in practical settings, given the existence of other well established, cost-free methods of monitoring and prescribing RT set-volume, such as stopping sets at a predetermined perceived number of repetitions in reserve (Helms et al. 2016).

## Conclusions

The present study expands on the previous velocity-based RT literature while providing several novel findings, specifically for free-weight exercises. While individual VL-%_repetitions_ relationships always provided greater goodness of fit compared to general relationships, both displayed high variability. Specifically, the results of the present study question the utility of using either general or individual VL-%_repetitions_ relationships to prescribe RT volume with a free-weight back squat exercise as (1) the agreement in the %_repetitions_ completed until reaching a given VL threshold across two consecutive testing sessions was not acceptable, regardless of the load used; and (2) the ability of both general and individual VL-%_repetitions_ relationships to predict %_repetitions_ in a subsequent testing session was poor and always led to prediction errors higher than 10%. Sex, training status, and history as well as personality traits did not affect the goodness of fit of general and individual VL-%_repetitions_ relationships as well as their prediction accuracy, thus suggesting potential generalisability of those findings among the resistance-trained population. Therefore, VL-%_repetitions_ relationships do not seem to provide any additional benefits compared to more traditional, free methods and hence should not be used for the purposes of monitoring and prescribing RT with a free-weight back squat exercise.

## Supplementary Information

Below is the link to the electronic supplementary material.

Supplementary file1 (DOCX 16 KB)

Supplementary file2 (DOCX 17 KB)

Supplementary file3 (DOCX 19 KB)

Supplementary file4 (DOCX 16 KB)

Supplementary file5 (DOCX 30 KB)

## Data Availability

The associated dataset for this study is available at the Open Science Framework repository (URL: https://osf.io/3yuvr/).

## Abbreviations

%1RM: One-repetition maximum
%_repetitions_: Percentage of performed repetitions out of the maximum possible in a set
CI: Confidence interval
IPIP: International personality item pool
LoA: Limits of agreement
LPT: Linear position transducer
MOVER: Method of variance estimates recovery
*R*^2^: Coefficient of determination
RSE: Residual standard error
RT: Resistance training
RTF: Repetitions to failure
TOST: Two one-sided tests
VL: Velocity loss
