# Supplementary material for: Velocity loss is a flawed method for monitoring and prescribing resistance training volume with a free-weight back squat exercise
Source: Eur J Appl Physiol. 2023 Feb 24;123(6):1343–57. doi: 10.1007/s00421-023-05155-x (PMC10192145; doi:10.1007/s00421-023-05155-x)
Supplement: Supplementary file 2 — Supplementary file2 (DOCX 17 KB) [file 421_2023_5155_MOESM2_ESM.docx]

Jukic et al. (2022). Velocity loss is a flawed method for monitoring and prescribing resistance training volume with free-weight exercises. *European Journal of Applied Physiology*. Email corresponding author: ivan.jukic@aut.ac.nz. Sport Performance Research Institute New Zealand (SPRINZ), Auckland University of Technology, Auckland, New Zealand

**Supplementary file II: Details on model specification and diagnostics**

For the analysis of agreement between percentages of the repetitions completed – with respect to the maximum possible – until reaching a given velocity loss threshold on two separate days, only first instances when a given velocity loss threshold was reached (i.e., 5% to 60% and more in 5% increments) were identified by expressing each repetition’s velocity (in each day and with a given load) as a percentage loss from the fastest repetition achieved in that day with a given load. Therefore, this analysis was limited to the instances when the participants managed to reach the same velocity loss threshold in both days (i.e., day 1 and day 2). For instance, number of repetitions could be compared (their differences or agreement) between the days if the person performed a certain number of repetitions until reaching 30% velocity loss with a given load, but not if the participant reached 30% velocity loss on day one, but “skipped” this threshold on day 2 (e.g., reaching 25% and 35% velocity loss with a given load but not 30%). Importantly, this did not affect modelling neither general nor individual relationships between velocity loss and the percentage of the completed repetitions where all the data could have been taken for the analysis (i.e., all participants’ data pooled for general models, and models for each participant separately for individual models).

A second order polynomial regression always yielded a better goodness of fit compared to simple liner regression for modelling the relationship between percentages of the repetitions completed – with respect to the maximum possible – and velocity loss. This was the case for both general and individual participants’ models. Therefore, predictive validity of only polynomial regression models was subsequently evaluated to reduce the number of unnecessary analyses and thus complexity of reporting. Importantly, while evaluating absolute differences between observed and predicted percentages of the completed repetitions – with respect to the maximum possible – several individual predictions yielded unrealistic values (i.e., predicted percentages of repetitions completed over 100, where the maximum number possible is 100). Therefore, these outliers were removed before examining the influential factors on prediction errors as they affected the stability of the models.

Since regression-based models can be sensitive to variables that are correlated, the variance inflation factors for all predictor parameters used in the linear mixed-effects model were inspected to check for multi-collinearity. For linear mixed-effects models, a Gaussian distribution was assumed, and the approximate normal distribution of model residuals was checked to confirm goodness of fit. To ensure the assumptions of the model were met, the plotted residuals were also checked to ensure homoscedasticity prior to utilising the results of the model. To validate the assumptions of the generalised mixed-effects model, a simulation-based approach based on examination of standardised residuals was used to test for uniformity of residuals, under and over dispersion, and outliers. None of the tests revealed issues with the models fit.
