# Supplementary material for: Velocity loss is a flawed method for monitoring and prescribing resistance training volume with a free-weight back squat exercise
Source: Eur J Appl Physiol. 2023 Feb 24;123(6):1343–57. doi: 10.1007/s00421-023-05155-x (PMC10192145; doi:10.1007/s00421-023-05155-x)
Supplement: Supplementary file 3 — Supplementary file3 (DOCX 19 KB) [file 421_2023_5155_MOESM3_ESM.docx]

Jukic et al. (2022). Velocity loss is a flawed method for monitoring and prescribing resistance training volume with free-weight exercises. *European Journal of Applied Physiology*. Email corresponding author: ivan.jukic@aut.ac.nz. Sport Performance Research Institute New Zealand (SPRINZ), Auckland University of Technology, Auckland, New Zealand

**Supplementary file III: Pairwise comparisons for significant, categorical variables with more than 2 levels for all outcomes of interest.**

Table 1. Pairwise comparisons with Holm-Bonferroni correction for the effects of loads on the coefficient of determination for the individual relationships between velocity loss and the percentage of the completed repetitions with respect to the maximum possible

| *contrast* | *estimate* | *SE* | *t.value* | *p.value* |
| --- | --- | --- | --- | --- |
| 70% / 80% | -0.02 | 0.01 | -2.40 | 0.03 |
| 70% / 90% | -0.03 | 0.01 | -2.62 | 0.03 |
| 80% / 90% | 0.00 | 0.01 | -0.22 | 0.82 |

Table 2. Pairwise comparisons with Holm-Bonferroni correction for the effects of loads on the predictive validity (i.e., absolute errors) of individual relationships between velocity loss and the percentage of the completed repetitions with respect to the maximum possible

| *contrast* | *estimate* | *SE* | *t.value* | *p.value* |
| --- | --- | --- | --- | --- |
| 70% / 80% | -0.72 | 0.62 | -1.16 | 0.24 |
| 70% / 90% | -6.11 | 0.80 | -7.64 | < 0.001 |
| 80% / 90% | -5.39 | 0.85 | -6.34 | < 0.001 |

Table 3. Pairwise comparisons with Holm-Bonferroni correction for the effects of training practices related to the loads used on the predictive validity (i.e., absolute errors) of individual relationships between velocity loss and the percentage of the completed repetitions with respect to the maximum possible

| *contrast* | *estimate* | *SE* | *t.value* | *p.value* |
| --- | --- | --- | --- | --- |
| 70% / 70 – 80% | 3.09 | 2.04 | 1.51 | 0.28 |
| < 70% / > 80% | 5.48 | 2.32 | 2.36 | 0.07 |
| 70 – 80% / > 80% | 2.39 | 1.72 | 1.39 | 0.28 |

Table 4. Pairwise comparisons with Holm-Bonferroni correction for the effects of loads on the probability of individual models not exceeding a prediction error of 10%

| *contrast* | *odds.ratio* | *SE* | *z.value* | *p.value* |
| --- | --- | --- | --- | --- |
| RTF70 / RTF80 | 1.02 | 0.14 | 0.14 | 0.88 |
| RTF70 / RTF90 | 0.53 | 0.09 | -3.77 | < 0.001 |
| RTF80 / RTF90 | 0.52 | 0.09 | -3.65 | < 0.001 |

Table 5. Pairwise comparisons with Holm-Bonferroni correction for the effects of training practices related to the loads used on the probability of individual models not exceeding a prediction error of 10%

| *contrast* | *odds.ratio* | *estimate* | *SE* | *z.value* | *p.value* |
| --- | --- | --- | --- | --- | --- |
| 70% / 70 – 80% | 1.24 | 3.09 | 0.36 | 0.75 | 0.45 |
| < 70% / > 80% | 1.96 | 5.48 | 0.65 | 2.03 | 0.13 |
| 70 – 80% / > 80% | 1.58 | 2.39 | 0.39 | 1.85 | 0.13 |
