# Supplementary material for: Velocity loss is a flawed method for monitoring and prescribing resistance training volume with a free-weight back squat exercise
Source: Eur J Appl Physiol. 2023 Feb 24;123(6):1343–57. doi: 10.1007/s00421-023-05155-x (PMC10192145; doi:10.1007/s00421-023-05155-x)
Supplement: Supplementary file 4 — Supplementary file4 (DOCX 16 KB) [file 421_2023_5155_MOESM4_ESM.docx]

Jukic et al. (2022). Velocity loss is a flawed method for monitoring and prescribing resistance training volume with free-weight exercises. *European Journal of Applied Physiology*. Email corresponding author: ivan.jukic@aut.ac.nz. Sport Performance Research Institute New Zealand (SPRINZ), Auckland University of Technology, Auckland, New Zealand

**Supplementary file IV: Absolute differences between predicted and observed percentages of the completed repetitions with respect to the maximum possible in the second testing session using the models established in the first testing session.**

|  | *general* | | | *individual* | | |  |
| --- | --- | --- | --- | --- | --- | --- | --- |
| *Load* | *Mean ε* | *Min ε* | *Max ε* | *Mean ε* | *Min ε* | *Max ε* | |
| 70% 1RM | 10.96 | 0.02 | 56.86 | 10.17 | 0.01 | 53.53 | |
| 80% 1RM | 10.49 | 0.01 | 66.96 | 10.74 | 0.04 | 63.16 | |
| 90% 1RM | 11.93 | 0.02 | 61.68 | 16.05 | 0.00 | 83.57 | |

Note. 1RM, one-repetition maximum; Mean ε, mean error; Min ε, minimal error; Max ε, maximal error.
