# Supplementary material for: Velocity loss is a flawed method for monitoring and prescribing resistance training volume with a free-weight back squat exercise
Source: Eur J Appl Physiol. 2023 Feb 24;123(6):1343–57. doi: 10.1007/s00421-023-05155-x (PMC10192145; doi:10.1007/s00421-023-05155-x)
Supplement: Supplementary file 5 — Supplementary file5 (DOCX 30 KB) [file 421_2023_5155_MOESM5_ESM.docx]

Jukic et al. (2022). Velocity loss is a flawed method for monitoring and prescribing resistance training volume with free-weight exercises. *European Journal of Applied Physiology*. Email corresponding author: ivan.jukic@aut.ac.nz. Sport Performance Research Institute New Zealand (SPRINZ), Auckland University of Technology, Auckland, New Zealand

**Supplementary file V: Percentage of the completed repetitions until reaching a given velocity loss thresholds across days, sex, training experience and relative strength levels**

|  |  | *Females* | | *Males* | | *Experience < 3 years* | | *Experience > 3 years* | | *Less strong* | | *Strong* | |
| --- | --- | --- | --- | --- | --- | --- | --- | --- | --- | --- | --- | --- | --- |
| *Load* | *VL* | *Day 1* | *Day 2* | *Day 1* | *Day 2* | *Day 1* | *Day 2* | *Day 1* | *Day 2* | *Day 1* | *Day 2* | *Day 1* | *Day 2* |
| 70% 1RM | VL5 | 18.97±10.26 | 20.66±13.38 | 19.1±8.65 | 21.43±11.18 | 18.99±10.53 | 20.29±11.28 | 19.1±8.17 | 22.06±12.36 | 21.61±10.79 | 20.06±12.45 | 17.77±8.2 | 21.64±11.64 |
| 70% 1RM | VL10 | 29.61±20.3 | 25.88±12.78 | 30.88±10.23 | 33.62±13.45 | 29.1±10.16 | 31.98±14.87 | 31.77±17.13 | 30.19±12.6 | 30.29±14.11 | 30.52±11.12 | 30.54±14.28 | 31.3±14.85 |
| 70% 1RM | VL15 | 38.68±10.42 | 41.99±16.08 | 41.81±14.92 | 44.95±13.67 | 42.15±15.6 | 44.87±13.82 | 39.84±12.13 | 43.1±15.19 | 37.09±16.84 | 51.17±15.83 | 42.6±12.27 | 40.18±12.29 |
| 70% 1RM | VL20 | 47.34±11.4 | 44.01±17.66 | 52.59±15.9 | 50.41±14.21 | 53.8±14.9 | 45.92±18.54 | 47.64±14.23 | 50.38±12.31 | 53.49±14.44 | 47.14±16.09 | 49.58±15.02 | 48.78±15.54 |
| 70% 1RM | VL25 | 54.41±17.21 | 49.98±17.06 | 60.5±14.76 | 61.77±10.45 | 61.41±16.44 | 58.91±14.66 | 55.68±15.04 | 56.24±13.98 | 59.01±17.18 | 56.24±12.87 | 57.87±15.24 | 58.19±15.01 |
| 70% 1RM | VL30 | 65.61±14.52 | 59.32±19.75 | 71.84±15.25 | 74.02±13.92 | 74.94±16.84 | 71.95±18.44 | 66.66±13.34 | 67.1±16.6 | 67.64±14.83 | 69.42±17.5 | 70.39±15.38 | 68.97±17.58 |
| 70% 1RM | VL35 | 76.35±14.27 | 74.16±15.56 | 78.77±13.23 | 76.01±14.46 | 77.75±12.49 | 74.07±16.58 | 78.2±14.79 | 76.35±13.24 | 77.28±13.52 | 76.75±13.2 | 78.26±13.66 | 74.39±15.78 |
| 70% 1RM | VL40 | 77.6±15.62 | 82.44±18.44 | 85.81±8.93 | 82.65±10.21 | 85.94±11.69 | 85.07±8.64 | 80.59±12.49 | 80.03±18.29 | 87.61±7.95 | 84.04±8.44 | 81.07±13.17 | 81.65±17.01 |
| 70% 1RM | VL45 | 86.08±11.39 | 85.38±10.45 | 93.76±6.02 | 90.22±5.79 | 92.24±5.1 | 88.99±8.93 | 88.58±11.83 | 89.14±5.99 | 89.12±4.29 | 90.28±9.71 | 90.48±10.97 | 88.71±6.53 |
| 70% 1RM | VL50 | 84.53±15.13 | 74.8±18.51 | 93.83±6.93 | 94.26±6.07 | 88.93±15.07 | 85.1±14.85 | 91.19±9.11 | 87.39±16.77 | 88.61±13.79 | 82.55±18.11 | 91.69±9.51 | 87.9±15.17 |
| 70% 1RM | VL55 | 88.72±4.38 | 89.24±12.51 | 93.27±7.39 | 96.59±5.92 | 92.16±8.78 | 100±0 | 91.69±5.88 | 91.21±9.8 | 93.73±10.85 | 100±0 | 91.31±5.79 | 93.24±9.32 |
| 70% 1RM | VL60≥ | 98.33±2.89 | 92.17±6.51 | 95.99±6.55 | 98.06±3.15 | 95.45±9.1 | 95.83±5.93 | 97.3±3.46 | 96.93±4.09 | 100±NA | 94.47±7.3 | 96.29±5.93 | 97.14±3.87 |
| 80% 1RM | VL5 | 29.97±11.72 | 28.71±16.36 | 26.38±11.27 | 29.51±19.26 | 25.12±11.09 | 26.62±13.42 | 30.32±11.43 | 32.19±22.57 | 23.14±9.4 | 21.89±15.21 | 30.13±11.8 | 31.9±18.85 |
| 80% 1RM | VL10 | 41.76±18.25 | 44.5±26.49 | 41.86±11.39 | 37.22±13.25 | 38.59±10.37 | 36.72±11.44 | 44.05±15.92 | 41.67±22.32 | 42.48±11.69 | 45.15±21.08 | 41.55±15.11 | 36.26±15 |
| 80% 1RM | VL15 | 46.51±14.15 | 45.94±26.53 | 45.35±13.21 | 47.74±9.72 | 46.18±13.55 | 48.71±20.24 | 45.14±13.5 | 45.61±15.31 | 48.02±13.79 | 48.16±20.56 | 43.9±13.03 | 46.7±16.93 |
| 80% 1RM | VL20 | 54.17±15.31 | 53.96±27.25 | 58±17.76 | 55.67±12.87 | 60.15±17.85 | 55.81±18.17 | 53.27±15.46 | 54.64±17.62 | 59.14±16.55 | 57.01±18.49 | 55.65±17.1 | 54.36±17.56 |
| 80% 1RM | VL25 | 62.34±12.21 | 61.37±21.33 | 68.55±16.25 | 66.95±11.82 | 67.39±12.3 | 68.94±12.15 | 65.29±17.34 | 63.15±16.24 | 70.87±11.45 | 68.97±7.52 | 63.98±16.24 | 64.5±16.07 |
| 80% 1RM | VL30 | 76.86±16.59 | 64.73±16.35 | 72.14±9.13 | 74.02±12.08 | 80.28±16.74 | 73.05±17.65 | 71.44±9.59 | 69.47±11.37 | 84.23±14.21 | 73.09±15.4 | 69.01±7.58 | 70.17±13.9 |
| 80% 1RM | VL35 | 88.38±6.78 | 76.32±15.94 | 80.28±11.2 | 75.92±16.69 | 84.57±11.65 | 83.4±15.03 | 79.92±8.78 | 67.8±13.19 | 88.08±5.54 | 83.2±5.5 | 81.37±11.25 | 73.86±17.56 |
| 80% 1RM | VL40 | 83.3±13.31 | 74.5±6.75 | 85.84±11.99 | 85.98±5.87 | 79.93±11.77 | 83.97±6 | 88.02±11.74 | 82.22±9.23 | 90.6±15.46 | 83.36±7.83 | 82.47±9.88 | 82.88±8.1 |
| 80% 1RM | VL45 | 86.44±5.18 | 86.58±5 | 90.51±9.99 | 92.33±10.77 | 82.51±7.57 | 92.56±7.29 | 95.21±5.57 | 88.77±10.06 | 79.7±8.09 | 86.65±4.74 | 92.76±6.89 | 90.55±9.67 |
| 80% 1RM | VL50 | 97.5±5.59 | 95.58±6.98 | 94.76±8.83 | 96.67±5.16 | 98.61±3.92 | 100±0 | 91.9±10.11 | 94.19±6.44 | 100±0 | 100±0 | 94.03±8.64 | 95.35±6.18 |
| 80% 1RM | VL55 | 100±NA | 100±0 | 95.96±5.58 | 97.11±4.96 | 90.9±NA | 97.78±4.96 | 97.78±4.96 | 98.48±3.72 | 90.9±NA | 100±0 | 97.78±4.96 | 97.47±4.71 |
| 80% 1RM | VL60≥ | 100±0 | NA±NA | 100±0 | 97.91±5.9 | 100±0 | 95.83±8.35 | 100±0 | 100±0 | 100±0 | 100±0 | 100±0 | 96.66±7.47 |
| 90% 1RM | VL5 | 38.1±14.55 | 53.7±11.6 | 55.03±20.43 | 64±27.74 | 51.69±20.42 | 61.56±14.33 | 42.72±19.21 | 57.77±36.73 | 40.72±14.6 | 73.35±9.4 | 52.52±21.32 | 55.73±24.46 |
| 90% 1RM | VL10 | 46.02±15.34 | 40.06±16.34 | 45.24±8.72 | 49.96±12.41 | 45.77±12.51 | 46.84±15.62 | 45.33±10.37 | 46.5±13.72 | 49.93±11.22 | 50.57±22.39 | 43.28±10.49 | 45.68±12.51 |
| 90% 1RM | VL15 | 61.73±14.85 | 51.38±15.08 | 62.81±21.22 | 61.57±18.91 | 66.77±16.62 | 56.38±24.41 | 58.49±20.4 | 57.01±11.66 | 65.57±15.03 | 57.92±24.24 | 61.82±19.65 | 56.2±14.67 |
| 90% 1RM | VL20 | 74.52±23.64 | 72.18±14.89 | 75±15.77 | 51.34±15.41 | 78±19.43 | 54.97±22 | 70.3±16.54 | 62.89±12.15 | 76.46±19.09 | 55.55±25.89 | 73.38±18.34 | 60.41±13.15 |
| 90% 1RM | VL25 | 61.67±15.65 | 77.43±10.22 | 75.09±18.36 | 71.49±18.14 | 74.42±17.28 | 68.97±14.74 | 69.5±19.4 | 77.07±16.57 | 64.28±10.57 | 77.5±3.54 | 74.05±20.21 | 72.97±16.8 |
| 90% 1RM | VL30 | 82.44±18.55 | 85±10 | 73.27±17.84 | 82.91±19.8 | 83.33±19.66 | 80.41±18.55 | 73.75±16.99 | 90±11.55 | 83.33±19.66 | 86.67±11.55 | 73.75±16.99 | 82.59±18.54 |
| 90% 1RM | VL35 | 81.25±8.84 | 83.33±28.87 | 90.17±10.97 | 82.3±15.17 | 87.58±11.02 | 73.2±23.61 | 89.88±11.42 | 88.88±12.55 | 84.9±14.37 | 75±35.36 | 89.98±10.32 | 84.51±15.38 |
| 90% 1RM | VL40 | 96.66±7.47 | 85.62±18.07 | 93.33±11.55 | 89.29±14.2 | 100±0 | 81.93±18.79 | 90.83±10.68 | 90.21±13.98 | 100±0 | 86.45±17.8 | 93.88±9.53 | 88.81±14.48 |
| 90% 1RM | VL45 | 100±0 | 100±0 | 97.14±6.4 | 90.34±13.3 | 95.23±8.26 | 92.49±12.28 | 100±0 | 100±0 | 95.23±8.26 | 100±0 | 100±0 | 90.34±13.3 |
| 90% 1RM | VL50 | 92.85±10.11 | 100±NA | 100±0 | 91.67±14.43 | 100±NA | 100±0 | 97.62±5.84 | 87.5±17.68 | 100±NA | 100±NA | 97.62±5.84 | 91.67±14.43 |
| 90% 1RM | VL55 | 100±NA | 100±0 | 100±NA | 100±0 | 100±NA | 100±0 | 100±NA | 100±0 | 100±NA | 100±NA | 100±NA | 100±0 |
| 90% 1RM | VL60≥ | - | - | 100±0 | 100±0 | 100±0 | 100±0 | 100±NA | 100±0 | 100±NA | 100±NA | 100±0 | 100±0 |

*Note.* 1RM, one-repetition maximum; VL, velocity loss; NA, not applicable (this happened when there were no data points for mean calculation or when only one data point was available for the calculation of the standard deviation).
